# Effects of low dietary calcium and lipopolysaccharide challenges on production performance, eggshell quality, and bone metabolism of laying hens

**DOI:** 10.3389/fphys.2024.1396301

**Published:** 2024-07-03

**Authors:** Xin Li, Victoria Anthony Uyanga, Hongchao Jiao, Xiaojuan Wang, Jingpeng Zhao, Yunlei Zhou, Haifang Li, Hai Lin

**Affiliations:** ^1^ Department of Animal Science and Technology, Shandong Provincial Key Laboratory of Animal Biotechnology and Disease Control and Prevention, Shandong Agricultural University, Key Laboratory of Efficient Utilization of Non-grain Feed Resources (Co-construction by Ministry and Province), Ministry of Agriculture and Rural Affairs, Taian, China; ^2^ College of Life Sciences, Shandong Agricultural University, Taian, China; ^3^ College of Chemistry, Shandong Agricultural University, Taian, China

**Keywords:** calcium, lipopolysaccharide, bone homeostasis, egg quality, aged laying hen

## Abstract

Dietary calcium supply is essential for bone development and egg production in laying hens. This study investigated the effects of low dietary calcium and lipopolysaccharide (**LPS**) induced immune challenge in aged laying hens. A total of thirty-two Hy-Line Brown laying hens at 80 weeks old with an average laying rate of 62% were randomly divided into two groups and fed a normal calcium diet (3.57% Ca, **NCA**) or low calcium diet (2.08% Ca, **LCA**). At 88 weeks, the experiment was designed using a 2 × 2 factorial arrangement, and hens were intraperitoneally injected with saline (**SAL**) or LPS (0.5 mg/kg, 0.5 mg/kg, or 1.5 mg/kg body weight) once every 48 h intervals over 5 days. Production performance, egg quality, and bone physiology were evaluated. Results showed that LPS challenge decreased the hen-day egg production, egg mass, and eggshell traits (*p* < 0.05), but increased (*p* < 0.05) the calcium content of the tibia compared to SAL-injected hens. LCA diet decreased (*p* < 0.05) the hen-day egg production, and eggshell traits such as weight, percentage, strength, and thickness compared to the NCA diet. LCA diet increased the serum alkaline phosphatase (**ALP**) activity (*p* < 0.01) and tibial expression of *ALP* (*p* < 0.05) compared to NCA diet. LPS injection suppressed both the serum ALP activity (*p* < 0.05) and tibial expression of *ALP* (*p* < 0.001) compared to SAL injection. Furthermore, LPS injection increased (*p* < 0.05) the expression of both pro and anti-inflammatory cytokines in the spleen and tibia. The expression of cathepsin K (***Cts K***) and matrix metalloproteinase 9 (***MMP-9***) were downregulated by LPS injection (*p* < 0.001). Broken and shell-less egg production and calcium content of eggshell, as well as tibial mRNA expression of osteocalcin (***Ocn***), tumor necrosis factor-alpha (***TNF-α***) and tartrate-resistant acid phosphatase (***TRAP***) were affected by the interaction (*p* < 0.05) of diet and injection. Therefore, this study demonstrated that to certain extents, low dietary calcium and LPS challenge dysregulated bone homeostasis and metabolism, with detrimental effects on the performance and eggshell quality of aged laying hens.

## 1 Introduction

In laying hens, calcium (**Ca**) deposition and mobilization in bone occurs daily to meet the requirements for egg production. The delicate balance between calcium and phosphorus deposition and resorption (i.e., bone synthesis and bone resorption) may be disrupted by intestinal malabsorption (Bielke et al., 2017), high output at the peak laying period (Whitehead, 2004), and immune challenge (Walsh et al., 2018). Bone health and the immune system are closely related since they both share a rich set of molecules and similar regulatory mechanisms (Takayanagi, 2007). There is sufficient evidence demonstrating that bone cells are primarily affected by various immune agents under normal and pathogenic conditions (Dar et al., 2018). Studies have also shown that inflammatory cytokines, mainly produced by T cells and B cells, can directly or indirectly regulate the functions of osteoclasts and osteoblasts (Takayanagi, 2007; Walsh et al., 2018). The osteoblasts and osteoclasts mainly mediate the process of bone formation and bone resorption (Horowitz, 1998). Osteoblasts promote self-produced osteoid mineralization by secreting alkaline phosphatase (**ALP**) and matrix vesicles (Knothe Tate et al., 2004). On the other hand, osteoclasts are involved in the hydrolysis of mineral composition and osteoid by generating acids and proteolytic enzymes (Zhang et al., 2011).

The pro-inflammatory cytokine, tumor necrosis factor-alpha (**TNF-α**) is considered a potent inducer of bone resorption and plays a crucial role in bone metabolism and inflammatory bone diseases (Amarasekara et al., 2015). It was shown that interleukin-17 (**IL-17**) inhibited the osteogenesis of rat calvarial osteoblast precursors, and reduced the expression of ALP and osteocalcin (**Ocn**) (Kim et al., 2014). In addition, bone cells can also regulate the function of immune cells (Tsukasaki and Takayanagi, 2019). Osteoblasts secrete multiple cytokines, including interleukin-1 (**IL-1**), interleukin-6 (**IL-6**), and interleukin-7, which regulate hematopoietic stem cells and support the differentiation of immune cells (Mercier et al., 2011). Inflammatory response via lipopolysaccharide (**LPS**) has been reported to affect bone homeostasis in broilers (Mireles et al., 2005) and laying hens (Nie et al., 2018; Akbari MoghaddamKakhki and Kiarie, 2021; Liu et al., 2022).

Conventionally, laying hens are reared up to 70 weeks with an average of 290 eggs during the first laying cycle (Ledur et al., 2000). However, some producers have considered that extending the laying period to more than 80 weeks to achieve up to 500 eggs can accrue significant economic benefits, increase profitability, and minimize the environmental impacts for sustainable production (Molnar et al., 2017; Molnar et al., 2018). Extended lay is associated with several problems such as a decline in egg production, a decrease in egg quality, shell quality, and bone quality, and a rise in birds’ health and welfare concerns (Molnar et al., 2017). To address these problems, improved genetics, along with appropriate nutrition strategy and management practices have been widely studied in aged laying hens (Xin et al., 2022). More so, it is necessary to understand the calcium metabolism of aged laying hens during the late laying stage.

Calcium supply is essential for bone development and eggshell formation in laying hens. Dietary supplementation of calcium is an important nutritional strategy for promoting the skeletal health of laying hens. Relative to hens fed normal calcium levels (3.69%), it was demonstrated that hens fed low calcium diets (1.56%) had greater bone pathological damage characterized by loss of bone mass and bone strength, lowered bone mineral content, increased micro-structural damage and a higher bone turnover (Jiang et al., 2019). The bone is an important source of calcium for eggshell formation, thus exploring bone homeostasis during the late egg-laying period will provide new ideas for prolonging the laying period. Bone metabolism is essential for laying hens because it is related to bone health and eggshell quality. However, the intestinal efficiency for calcium absorption decreases in aged hens, thus limiting the calcium supply to the blood, which results in poor eggshell quality (Molnar et al., 2018). The decrease in blood calcium does not only affect the calcium supply for eggshell formation but directly impacts the metabolism and adjustment for calcium in the bone (Gu et al., 2021). Therefore, low calcium levels can disrupt normal bone metabolism, and thus affect eggshell formation.

Since bone homeostasis is established by the metabolic balance between osteoblasts and osteoclasts, it is worth understanding the underlying changes that occur during immune challenge and low calcium conditions. Therefore, this study investigated the interaction of dietary calcium levels and LPS challenge on the production performance, egg quality, bone immune function, and metabolism-related genes in aged laying hens.

## 2 Material and methods

This research was performed in accordance with the “Guidelines for Experimental Animals” of the Ministry of Science and Technology (Beijing, P. R. China), and the study protocols were approved by the Institutional Animal Care and Use Committee (No. 2020002) of the Shandong Agricultural University, China.

### 2.1 Experimental animals and management

Hy-Line Brown laying hens at 78 weeks old (n = 99) were randomly divided into two groups and fed a normal calcium diet, with a 2-week adaptation period before the start of the experiment. Hens were raised in battery cages that housed two birds per cage (60 cm length × 45 cm width × 75 cm height). The housing temperature and relative humidity were maintained at 23°C ± 2°C and 65% ± 3%, respectively, while the photoperiod was 16 h light and 8 h dark. Each cage was equipped with 1 nipple drinker and 1 feeding trough. All hens had free access to feed and water throughout the experimental period. Thirty eggs were collected from each group at 78 weeks of age for eggshell quality measurement.

At 80 weeks of age, 32 hens with similar body weight (1.98 ± 0.02 kg) and an average laying rate of 62% were selected and housed two hens per cage. The hens were divided into two groups and fed either the normal calcium diet (3.57% Ca, **NCA**) or a low calcium diet (2.08% Ca, **LCA**) until they reached 88 weeks of age. The formulation of NCA diet adhered to the guidelines of the Feeding Standard of Chickens (NY/T33-2004, China), and the dietary Ca level of LCA diet was based on a previous report (Table 1) (Jiang et al., 2019). Eggs were collected daily in the morning and evaluated weekly on an individual basis to determine the laying rate (%). Three or four eggs were collected from each cage (30 eggs in each group) on three consecutive days at 83 and 87 weeks, respectively.

**TABLE 1:** Ingredients and nutrient composition of experimental diets.

Ingredient, %	NCA^a^	LCA^a^
Corn	55.40	58.27
Soybean meal (43 CP%)	23.83	22.76
Wheat bran	6.24	7.50
Soybean oil	2.22	1.22
Limestone	9.29	2.73
Calcium hydrogen phosphate	1.22	1.20
NaCl	0.34	0.34
Zeolite powder	1.00	5.50
Lysine (99%)	-	0.01
Methionine (98%)	0.11	0.11
Threonine (98%)	-	0.01
Choline chloride (50%)	0.10	0.10
Premix^b^	0.25	0.25
Calculated nutrient levels		
Metabolizable energy, MJ/kg	11.10	11.10
Crude protein, %	16.00	16.00
Calcium, %	3.61	1.32
Lysine, %	0.750	0.750
Methionine, %	0.368	0.366
Methionine + Cystine, %	0.650	0.650
Threonine, %	0.641	0.641
Tryptophan, %	0.213	0.211
Availablephosphorus, %	0.350	0.350
Analyzed nutrient levels		
Calcium, %	3.57	2.08

^a^ NCA: normal calcium diet; LCA: low calcium diet.

^b^ The vitamin and mineral premix provides the following quantities per kilogram of diet: vitamin A, 12,000 IU; vitamin D3, 2,400 IU; vitamin K, 0.75 mg; vitamin E, 7.5 IU; cholecalciferol, 2,400 IU; riboflavin, 3.75 mg; niacin, 30 mg; pantothenic acid, 3.3 mg; biotin, 0.15 mg; folic acid, 0.375 mg; Thiamine, 1.2 mg; Pyridoxine, 4.5 mg; Vitamin B12, 0.006 mg; Fe, 60 mg; Se, 0.3 mg; Cu, 10 mg; Zn, 80 mg; I, 0.35 mg; Mn, 60 mg.

**FIGURE 1:**
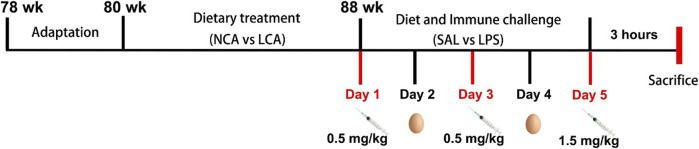
Diagram depicting the experimental schedule. NCA: Normal calcium diet (3.57% Ca); LCA: Low calcium diet (2.08% Ca). SAL: Saline; LPS: Lipopolysaccharide.

At 88 weeks of age, hens were individually reared and grouped into a 2 × 2 factorial arrangement for a five-day trial and were subjected to either normal calcium diet + saline (**NCA + SAL**), normal calcium diet + LPS (**NCA + LPS**), low calcium diet + saline (**LCA + SAL**), or low calcium diet + LPS (**LCA + LPS**). Thus, the experiment was arranged as 4 treatments, each consisting of 8 replicates. A cage with one hen was considered as an experimental unit. Laying hens were intermittently injected intraperitoneally with either sterile saline, or LPS (derived from *Escherichia coli*, L2880; Sigma-Aldrich, St. Louis, MO, USA) at doses of 0.5, 0.5, or 1.5 mg/kg body weight at 11:00 a.m. on days 1, 3, and 5 of week 88 (every 48-h interval) (Nie et al., 2018). Performance, egg production, and egg quality data were collected during the trial. All data were collected on an individual basis. Feed intake was recorded weekly, and egg weights and number of eggs laid from each chicken were recorded daily to determine the daily feed intake (g), hen-day egg production (%), and average egg weight (g). Egg mass was determined by multiplying the average egg weight by egg numbers and then dividing it by the number of days. Feed conversion ratio was calculated as grams of feed: grams of egg mass produced. The diagram depicting the experimental schedule experimental design is shown in Figure 1.

### 2.2 Sample collection

Five eggs from each hen and in total 40 eggs from each treatment were collected on the second and fourth days for eggshell quality assessment. On the fifth day of trial, a blood sample was collected at 14:00 p.m., 3 h after LPS or saline injection, from a wing vein of all the 32 experimental hens. Serum samples were obtained after centrifugation at 1,500 × g for 15 min at 4°C and stored at −20°C for further analysis. After blood collection, birds were sacrificed by exsanguination after cervical dislocation (Close et al., 1997; Sun et al., 2020). The spleen and right tibia were collected and immediately snap-frozen in liquid nitrogen, then stored at −80°C for further analysis. The left tibia was isolated and stored at −20°C for bone quality measurement. The distal right femur was obtained and fixed in 10% neutral buffered formalin for histological analysis.

### 2.3 Egg quality evaluation

The egg weight was measured using the egg multi-tester (EMT-5200, Robotmation, Japan), and eggshell strength was measured with an egg force reader (EFG-0503, Robotmation Co., Ltd., Tokyo, Japan). Eggshell thickness was measured with an eggshell thickness tester (ETG-1061, Robotmation Co., Ltd.) at three different locations (air cell, equator, and sharp end) on the egg, and the average was computed. Eggs were cracked, and the shells were cleaned, dried at 25°C for 12 h, and then weighed. The eggshell percentage was calculated using the formula: (eggshell weight/egg weight) × 100%.

### 2.4 Serum biochemical parameters

Serum ionized calcium and phosphorus levels were analyzed using an automatic biochemical analyzer (7170A, Hitachi, Japan) and corresponding kits provided by Maccura (Sichuan, China). Activities of alkaline phosphatase (ALP) (A059-2, Nanjing Jiancheng Bioengineering Institute, Nanjing, China) and tartrate-resistant acid phosphatase (**TRAP**) (P0332, Beyotime Biotechnology, Shanghai, China) were assessed with specific commercial assay kits, following the manufacturer’s guidelines.

### 2.5 Tibia physical parameters

Bone mineral density (**BMD**) of the left tibia was measured using the Dual-energy X-ray absorptiometry device (InAlyzer; MEDIKORS Inc., Gyeonggi-Do, Korea). Bone breaking strength of the left tibia was measured using a 3-point bending test with a microcomputer-controlled electronic universal testing machine (Jinan Shijin Group Co., Ltd., China). The bone was positioned on two supports with a 3 cm span, and a load of 1,000 N was applied at the midpoint of each bone at a loading speed of 2 mm/min until fracture. The computerized monitor recorded the deformation curve, with the peak load indicating the breaking strength in Newton. Left tibia samples were treated with a mixture of alcohol and benzene (2:1) for 96 h to get rid of fat and were then dried at 105°C to a constant weight. The defatted bone index was calculated as defatted bone weight (g)/body weight (kg) × 100.

### 2.6 Calcium and phosphorus content in diet, tibia, and eggshell

Eggshell and defatted left tibia samples were ground into a powder with a pulverizer. About 0.5 g of diet, tibia, and eggshell samples were weighed into a crucible and carbonized using an induction cooker. Samples were ashed in a muffle furnace at 550°C for 6 h, then dissolved in a mixture of hydrochloric acid and concentrated nitric acid, and transferred to a 100 mL volumetric flask after boiling to prepare a sample decomposition solution. The Ca content was measured using potassium permanganate titration, and the P content was determined by the ammonium molybdate spectrophotometric method, as previously reported (Song et al., 2022).

### 2.7 Bone histological analysis

The fully fixed distal femurs were incubated in an EDTA decalcifying solution (E1171; Beijing Solarbio Science & Technology Co., Ltd., China) at room temperature for about 2 weeks. Decalcified femur samples were dehydrated in a graded series of ethanol and routinely embedded in paraffin. The samples were then cut into 3 μm sections for hematoxylin-eosin (**HE**) staining and Goldner Trichrome staining (G3550; Solarbio, China) according to the manufacturer’s instructions. The slides were stained and visualized under an optical microscope (CK-40, Olympus, Tokyo, Japan). Images were taken of each section, and ImageJ software was used to measure the tissue area (**T.Ar**), trabecular area (**Tb.Ar**). Trabecular bone volume/tissue volume (**BV/TV**) was calculated as BV/TV = Tb.Ar/T.Ar × 100 (%).

### 2.8 Total RNA extraction and real-time quantitative PCR

Spleen samples were homogenized using a grinder (Genenode, China), while tibia samples were ground to powder using liquid nitrogen with a mortar. Total RNA was obtained from the spleen and tibia using Trizol reagent (Invitrogen, USA). The concentration and purity of the RNA were verified from OD_260/280_ readings (Ratio ≈1.75–2.01) using spectrophotometry (Eppendorf, Germany). According to the manufacturer’s instructions, reverse transcription was performed using total RNA (1,000 ng) for first-strand cDNA synthesis with the Transcriptor First Strand cDNA Synthesis Kit (Roche, Germany). The cDNA was amplified in a 20 μL reaction system containing 0.2 μM of each specific primer (Sangon, China) and the SYBR Green master mix (Roche, Germany). RT-qPCR was performed at the ABI QuantStudio 5 PCR machine (Applied Biosystems; Thermo, USA). Primers used for RT-qPCR were designed with Primer 5.0 software and synthesized by Sangon Biotech (Shanghai, China) according our previous work (Song et al., 2022), and primer sequences are shown in Table 2. The melting curves were checked to guarantee the specificity of amplification products. Data were analyzed using the method of 2^−ΔΔCT^, and GAPDH was used to normalize the data as an internal control. The mRNA expression level of the NCA + SAL group was used as a calibrator. The expressions of genes tumor necrosis factor α (*TNF-α*), interleukin 1β (*IL-1β*), interleukin 6 (*IL-6*), interleukin 17 (*IL-17*), interleukin 10 (*IL-10*), interferon γ (IFN-γ), alkaline phosphatase (*ALP*), osteocalcin (*Ocn*), and fibroblast growth factor 23 (*FGF23*) were determined.

**TABLE 2:** Primers used for RT-qPCR.

Gene^a^	GenBank ID	Forward primers (5′-3′)	Reverse primers (5′-3′)	Product size (bp)
*TNF-α*	MF000729.1	GTA​ACG​GCG​TGG​TGC​TGA​GAA​G	TCC​TCG​GAG​AAG​CGG​CTG​AC	142
*IL-1β*	NM_204524.1	CAG​AAG​AAG​CCT​CGC​CTG​GAT​TC	GCC​TCC​GCA​GCA​GTT​TGG​TC	133
*IL-6*	NM_204628.1	GAG​GTT​GGG​CTG​GAG​GAG​GAG	TCT​CGC​ACA​CGG​TGA​ACT​TCT​TG	130
*IL-17*	NM_204460.1	CGA​TGA​GGA​CCA​CAA​CCG​CTT​C	TGT​TTG​ATG​GGC​ACG​GAG​TTG​AC	117
*IL-10*	NM_001004414.2	CAG​CAC​CAG​TCA​TCA​GCA​GAG​C	GCA​GGT​GAA​GAA​GCG​GTG​ACA​G	94
*IFN-γ*	NM_205149.1	CTC​GCA​ACC​TTC​ACC​TCA​CCA​TC	CAG​GAA​CCA​GGC​ACG​AGC​TTG	122
*ALP*	NM_205360.1	CAC​GGC​GTC​GAT​GAG​CAG​AAC	AGC​AGA​GGA​GGA​GGA​GGA​GGA​G	148
*Ocn*	NM_205387.4	GCA​GGC​AGA​AGC​GGC​ACT​AC	CAG​CTC​ACA​CAC​CTC​TCG​TTG​G	89
*FGF23*	XM_425663.4	AGC​CAA​GAG​GAC​TGT​GTG​TTC​AAC	ACT​GGG​AGT​ACG​GTG​GTG​GAT​TC	145
*TRAP*	XM_015302697.2	TGC​TGG​CTT​TGG​GCG​ATA​ACT​TC	GCC​GTG​GTG​GTC​GTG​GTT​TC	149
*Cts K*	NM_204971.2	GAA​GGC​AAC​GAG​AAG​GCT​CTG​AAG	AGA​ACT​GGA​AGG​AGG​GCA​GAC​TG	91
*MMP-9*	NM_204667.1	ACC​TGG​ACC​GTG​CCG​TGA​TAG	CTG​CCT​CGC​CGC​TGT​AAA​TCT​G	102
*GAPDH*	NM_204305.1	CAG​AAC​ATC​ATC​CCA​GCG​TCC​AC	CGG​CAG​GTC​AGG​TCA​ACA​ACA​G	134

^a^ *TNF-α*, tumor necrosis factor alpha; *IL-1β*, interleukin 1 beta; *IL-6*, interleukin 6; *IL-17*, interleukin 17; *IL-10*, interleukin 10; *IFN-γ*, interferon gamma; *ALP*, alkaline phosphatase; *Ocn*, osteocalcin; *FGF23*, fibroblast growth factor 23; *TRAP*, tartrate-resistant acid phosphatase; *Cts K*, Cathepsin K; *MMP-9*, matrix metallopeptidase 9; *GAPDH*, glyceraldehyde-phosphate dehydrogenase.

### 2.9 Statistical analysis

Statistical analysis was conducted using the PROC GLM procedure of SAS software (version 9.3, SAS Institute Inc., Cary, NC) with each hen as a replicate. Prior to LPS challenge, data was subjected to One-way ANOVA to analyze the main effect of diet (NCA vs. LCA). Following LPS challenge, two-way ANOVA was used to analyze the significant main effects of diet (NCA vs. LCA), injection (Saline vs. LPS) and their interaction effect (diet × injection). Duncan’s Multiple Range Test was used for mean comparisons where the treatment effect was significant. Treatment differences were considered significant at *p <* 0.05 and the tendency towards significance was noted at 0.05 < *p* ≤ 0.10.

## 3 Results

### 3.1 Production performance and egg quality traits

All hens were fed a normal diet during the adaptation period, and eggshell quality did not differ (*p* < 0.05, Table 3) significantly between the two groups.

**TABLE 3:** Effects of dietary calcium levels on the laying rate and eggshell quality of aged laying hens prior to LPS challenge.^a^.

Items	NCA	LCA	SEM	*p*-value
Laying rate (%)				
83 wk	69.64^a^	58.93^b^	3.34	0.035
84 wk	71.43^a^	59.82^b^	3.26	0.021
85 wk	77.68^a^	66.07^b^	3.84	0.045
86 wk	76.79^a^	65.18^b^	3.30	0.022
87 wk	66.67	60.42	3.40	0.201
Eggshell weight (g)				
78 wk^b^	5.86	5.84	0.12	0.919
83 wk	5.85	5.86	0.12	0.965
87 wk	6.50^a^	5.69^b^	0.08	<. 001
Eggshell percentage (%)				
78 wk^b^	9.30	9.01	0.16	0.194
83 wk	9.50	9.28	0.20	0.424
87 wk	10.08^a^	8.83^b^	0.14	<. 001
Eggshell strength (kg. f)				
78 wk^b^	3.21	3.10	0.16	0.650
83 wk	3.25^a^	2.68^b^	0.10	<. 001
87 wk	3.40^a^	2.70^b^	0.13	0.002
Eggshell thickness (mm)				
78 wk^b^	0.32	0.33	0.01	0.544
83 wk	0.30^a^	0.29^b^	0.00	0.028
87 wk	0.32^a^	0.29^b^	0.01	<. 001

NCA: Normal calcium diet (3.57% Ca); LCA: Low calcium diet (2.08% Ca); SEM: standard error of mean.

^a^ with each cage as replicate (n = 8).

^b^ n = 30.

^a-b^Means with different alphabetical superscripts within each row are significantly different (*p* < 0.05).

Prior to LPS challenge, laying hens were fed dietary treatments from 80 to 88 weeks of age. Table 3 shows that the laying rate was significantly reduced (*p* < 0.05) by the LCA dietary treatments during weeks 83–86. The eggshell strength and eggshell thickness were lower in the LCA compared to the NCA diet at 83 weeks. This effect was further pronounced at week 87, as LCA-fed hens had lesser eggshell weight and percentage (*p* < 0.001), decreased eggshell strength (*p* < 0.01), and reduced eggshell thickness (*p* < 0.001) than those fed NCA diets.

During LPS challenge period, LPS injection reduced hen-day egg production and egg mass (*p* < 0.001, Table 4). In contrast, LPS treatment had no effect (*p* > 0.05) on body weight, egg weight, and feed conversion ratio. The average daily feed intake, however, tended to be decreased by LPS injection (*p* = 0.054), compared to SAL-treated hens. There was a significant interaction of diet × LPS (*p* < 0.05) on the percentage of broken and shell-less egg and the LCA + LPS group produced more broken and shell-less eggs, compared to other groups. Compared to the NCA diet, LCA treatment decreased (*p* < 0.05) hen-day egg production and egg mass (Table 4), whereas had no influence (*p* > 0.05) on body weight, average daily feed intake, egg weight, and feed conversion ratio.

**TABLE 4:** Effects of dietary calcium levels and LPS challenge on the performance and eggshell quality of aged laying hens^1^.

	NCA		LCA		SEM	NCA	LCA	SAL	LPS	*p*-value		
	SAL	LPS	SAL	LPS						Diet	LPS	Diet×LPS
*Performance*												
Body weight (kg)	1.92	1.88	1.91	1.85	0.08	1.91	1.88	1.92	1.87	0.775	0.542	0.908
Average daily feed intake (g)	113.0	90.98	117.34	71.45	15.91	101.99	94.39	115.17	81.21	0.642	0.054	0.467
Hen-day egg production(%)	77.51	53.33	59.38	30.00	8.10	65.42^m^	44.69^n^	68.44^x^	41.67^y^	0.025	0.006	0.753
Broken and shell-less egg production (%)	-	8.33^b^	4.71^b^	35.42^a^	4.12	0.04	0.20	0.02	0.22	0.003	<.001	0.017
Average egg weight (g)	64.22	61.50	61.85	62.37	1.37	62.86	62.11	63.04	61.94	0.596	0.438	0.260
Egg mass (g)	52.13	33.01	36.75	18.83	5.24	42.57^m^	27.79^n^	44.44^x^	25.92^y^	0.020	0.006	0.916
Feed conversion ratio (feed, g/egg mass, g)	2.14	3.61	3.47	4.38	1.10	2.91	3.92	2.84	3.40	0.378	0.316	0.826
*Eggshell Quality*												
Eggshell weight (g)	6.07	5.98	5.69	5.13	0.26	6.03^m^	5.41^n^	5.88	5.56	0.023	0.217	0.369
Eggshell percentage (%)	9.63	9.34	9.15	8.32	0.36	9.48^m^	8.74^n^	9.39	8.83	0.047	0.132	0.460
Eggshell strength (kg. f)	3.53	3.47	2.81	2.81	0.27	3.50^m^	2.81^n^	3.17	3.14	0.017	0.909	0.926
Eggshell thickness (mm)	0.33	0.30	0.31	0.28	0.01	31.70^m^	29.41^n^	31.93^x^	29.18^y^	0.040	0.015	0.650
Ca content of eggshell (%)	35.21^a^	30.29^c^	32.58^b^	32.79^b^	0.70	32.75	32.69	33.89	31.54	0.932	0.002	0.001
P content of eggshell (%)	14.75	14.06	14.12	11.59	0.66	14.40^m^	12.85^n^	14.43^x^	12.83^y^	0.026	0.021	0.174

NCA: Normal calcium diet (3.57% Ca); LCA: low calcium diet (2.08% Ca); SAL: saline; LPS: lipopolysaccharide; Ca: Calcium; P: phosphorus; SEM: standard error of mean.

^a^ with each cage as replicate (n = 8).

^a-c^Means with different alphabetical superscripts within the same row differ significantly (*p* < 0.05).^n, m^Means with different alphabetical superscripts within column NCA, and LCA, differ significantly (*p* < 0.05).^x, y^Means with different alphabetical superscripts within column SAL, and LPS, differ significantly (*p* < 0.05).

Compared to NCA-hens, LCA treatment decreased (*p <* 0.05) eggshell weight, eggshell percentage, eggshell strength, eggshell thickness, and P content of eggshell (Table 4). LPS challenge significantly reduced (*p <* 0.05) the eggshell thickness and P content of eggshell (Table 4). In contrast, LPS treatment had no significant (*p* > 0.05) influence on eggshell weight, percentage, and strength. There was a significant interaction of diet × LPS on the Ca content of eggshell (*p* < 0.01, Table 4). The NCA + SAL group had the highest Ca content, whereas the NCA + LPS group had the lowest Ca content of eggshells (*p* < 0.01). In contrast, LPS treatment had no influence (*p* > 0.05) on Ca content in LCA groups.

### 3.2 Serum parameters

Serum concentrations of total Ca and TP, and TRAP activity were not altered by diet, LPS, or their interaction between diet and injection (*p* > 0.05, Table 5). However, serum ALP activity was significantly increased (*p* < 0.01) by the LCA diet compared to the NCA diet. In addition, the LPS challenge significantly decreased (*p* < 0.001) serum ALP activity compared to the SAL-injected hens.

**TABLE 5:** Effects of dietary calcium levels and LPS challenge on the serum biochemical parameters of aged laying hens.

	NCA		LCA		SEM	NCA	LCA	SAL	LPS	*p*-value		
	SAL	LPS	SAL	LPS						Diet	LPS	Diet× LPS
Ionized Ca (mmol/L)	5.71	5.69	5.70	4.94	0.27	5.70	5.32	5.71	5.32	0.174	0.163	0.179
Inorganic P (mmol/L)	1.05	1.17	1.30	1.23	0.11	1.11	1.27	1.17	1.20	0.168	0.819	0.428
ALP (U/L)	146.40	62.90	230.10	119.40	21.50	104.67^n^	174.79^m^	188.29^x^	91.169^y^	0.003	<.001	0.533
TRAP (U/L)	49.10	46.60	49.30	35.50	8.50	47.84	42.42	49.19	41.07	0.527	0.346	0.509

NCA: Normal calcium diet (3.57% Ca); LCA: Low calcium diet (2.08% Ca); SAL: saline; LPS: lipopolysaccharide; ALP: alkaline phosphatase; TRAP: tartrate-resistant acid phosphatase; SEM: standard error of mean, n = 8.^n, m^Means with different alphabetical superscripts within column NCA, and LCA, are significantly different (*p* < 0.05).^x, y^Means with different alphabetical superscripts within column SAL, and LPS, are significantly different (*p* < 0.05).

### 3.3 Bone parameters

Neither diet nor the diet × LPS interaction significantly affected (*p* > 0.05) the defatted bone index, Ca and P content of the tibia, BMD, or bone breaking strength (Table 6). In addition, it was observed that the LPS injection significantly increased (*p* < 0.05, Table 6) the Ca content of the tibia compared to SAL-hens.

**TABLE 6:** Effects of dietary calcium levels and LPS challenge on the tibia bone parameters of aged laying hens.

	NCA		LCA		SEM	NCA	LCA	SAL	LPS	*p*-value		
	SAL	LPS	SAL	LPS						Diet	LPS	Diet×LPS
Defatted bone index (g/kg)	3.70	3.74	3.59	4.00	0.17	3.72	3.80	3.65	3.87	0.673	0.200	0.284
Ca content (%)	16.16	18.61	17.31	17.75	0.58	17.39	17.53	16.74^x^	18.18^y^	0.804	0.018	0.092
P content (%)	8.77	9.32	9.02	9.07	0.26	9.05	9.05	8.89	9.20	0.996	0.261	0.357
BMD (g/cm^2^)	0.29	0.31	0.28	0.29	0.02	0.30	0.29	0.29	0.30	0.582	0.502	0.803
Bone breaking strength (N)	158.1	153.5	164.2	148.8	18.60	155.82	156.56	161.20	151.19	0.968	0.596	0.776

NCA: Normal calcium diet (3.57% Ca); LCA: Low calcium diet (2.08% Ca); SAL: saline; LPS: lipopolysaccharide; Ca: Calcium; P: phosphorus; BMD: bone mineral density; SEM: standard error of mean, n = 8. ^x, y^Means with different alphabetical superscripts within column SAL, and LPS, are significantly different (*p* < 0.05).

**FIGURE 2:**
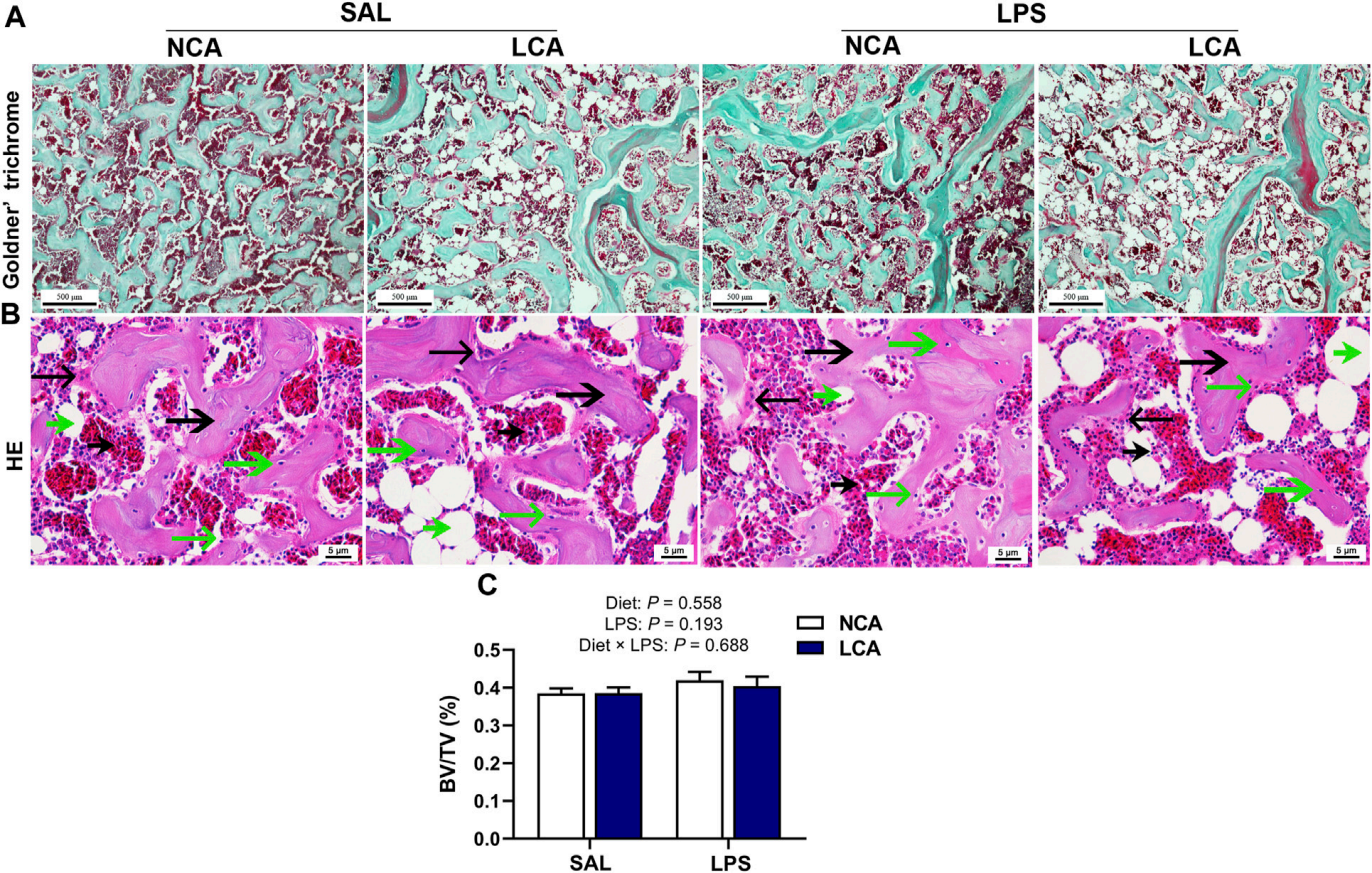
Effects of dietary calcium levels and LPS challenge on the bone microstructure of aged hens. NCA: Normal calcium diet (3.57% Ca); LCA: Low calcium diet (2.08% Ca). SAL: Saline; LPS: Lipopolysaccharide. **(A)** Goldner’ Trichrome staining of the distal femur (scale bar: 500 μm); **(B)** HE staining of the distal femur (scale bar: 5 μm); **(C)** Trabecular bone volume/tissue volume (BV/TV)
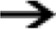
trabecular bone; 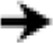
connective tissue cells; 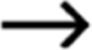
osteoclast; 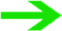
osteocyte; 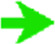
adipocyte
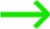
osteoblast.

Furthermore, Goldner’s trichrome staining of the distal femur showed that the mineralized bones were stained green while the osteoid or collagen was stained red (Figure 2A). HE staining showed the trabecular bone and connective tissue cells (Figure 2B). Compared with SAL treatment, LPS treatment showed more adipocytes with large volume (Figure 2B). However, there was no significant difference in trabecular bone volume/tissue volume among treatments (Figure 2C).

### 3.4 mRNA expression of inflammation-related genes in the spleen and tibia

**FIGURE 3:**
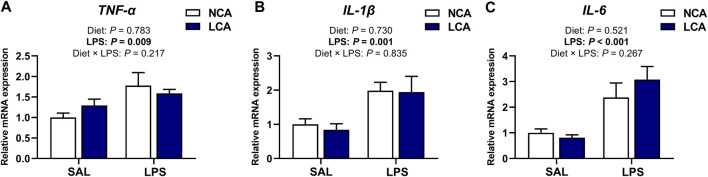
Effects of dietary calcium levels and LPS challenge on the relative mRNA expression of splenic inflammation-related genes in aged laying hens. NCA: Normal calcium diet (3.57% Ca); LCA: Low calcium diet (2.08% Ca). SAL: Saline; LPS: Lipopolysaccharide. **(A)** *TNF-α*; **(B)** *IL-1β*; **(C)** *IL-6.* Data are represented as mean ± standard error of mean (n = 8).

In the spleen, mRNA expression levels of *TNF-α*, *IL-1β,* and *IL-6* were not affected by the main effect of diet (*p* > 0.05) or by diet × LPS interaction (*p >* 0.05, Figure 3). However, the expression of *TNF-α* (*p* < 0.01), *IL-1β* (*p* < 0.01), and *IL-6* (*p* < 0.001) was upregulated in the LPS-challenged groups compared to the SAL-injected hens (Figures 3A–C).

**FIGURE 4:**
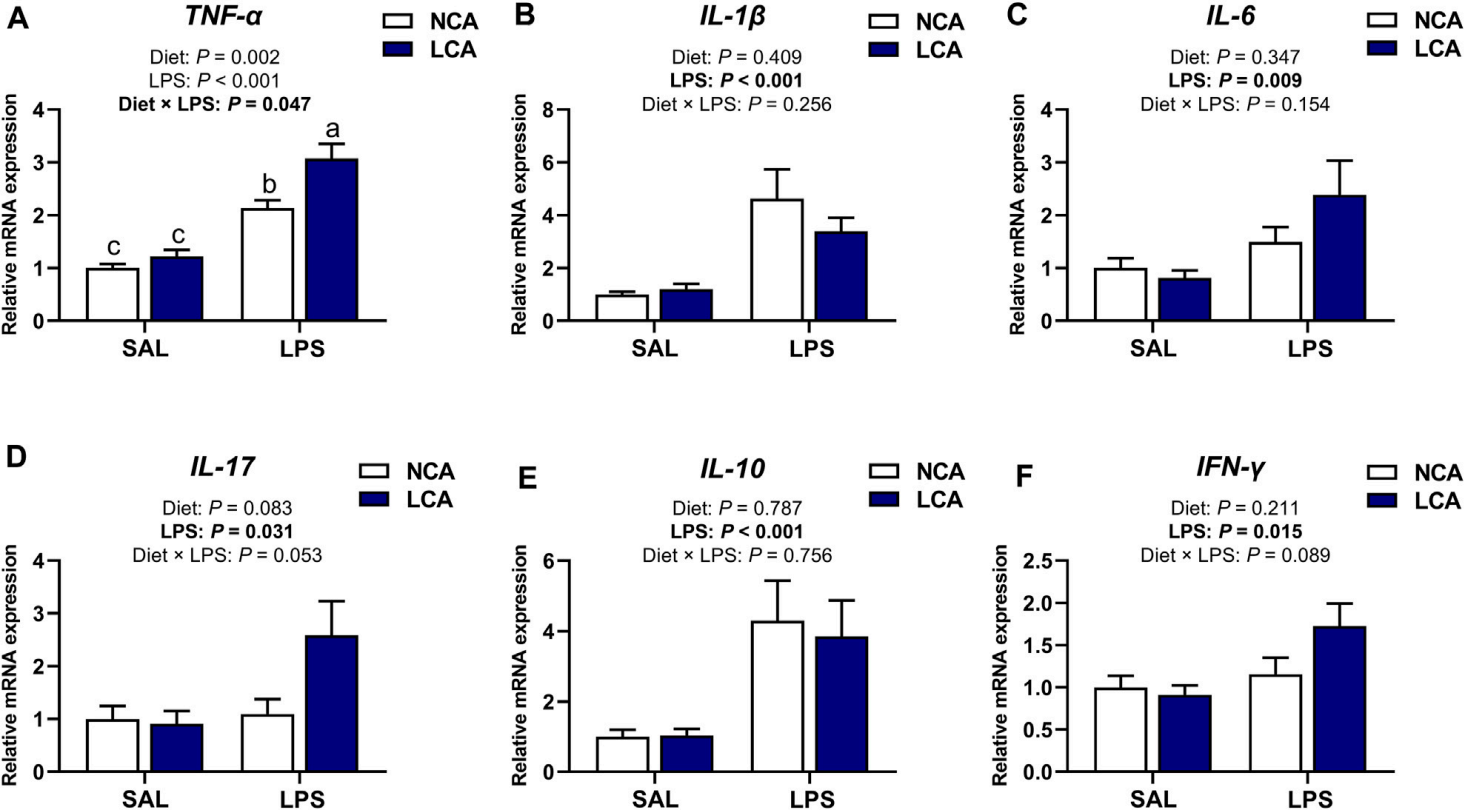
Effects of dietary calcium levels and LPS challenge on the relative mRNA expression of tibial inflammation-related genes in aged laying hens. NCA: Normal calcium diet (3.57% Ca); LCA: Low calcium diet (2.08% Ca). SAL: Saline; LPS: Lipopolysaccharide. **(A)** *TNF-α*; **(B)** *IL-1β*; **(C)** *IL-6*; **(D)** *IL-17*; **(E)** *IL-10*; **(F)** *IFN-γ.* Data are represented as mean ± standard error of mean (n = 8).

The tibial mRNA expression level of *TNF-α* (Figure 4A) was significantly affected by the diet × LPS interaction (*p* < 0.05) and the LPS-induced increment of *TNF-α* expression was further increased by LCA treatment. The mRNA abundances of *IL-1β* (*p* < 0.001), *IL-6* (*p* < 0.01), *IL-17* (*p* < 0.05)*, IL-10* (*p* < 0.001), and *IFN-γ* (*p* < 0.05) were upregulated in the tibia of LPS-challenged hens compared to SAL-hens (Figure 4). Alongside this, there was a tendency for *IL-17* (Figure 4D; *p* = 0.083) expression to be upregulated in the LCA dietary treatment compared to the NCA diet. The expression of *IL-17* (*p* = 0.053) and *IFN-γ* (Figure 4F; *p* = 0.089) showed a tendency to be affected by the interaction effect of diet and LPS.

### 3.5 mRNA expression of tibial osteoblast metabolism-related genes

**FIGURE 5:**
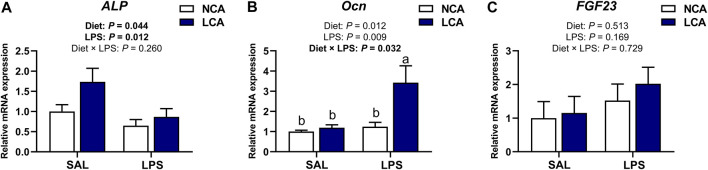
Effects of dietary calcium levels and LPS challenge on the relative mRNA expression of tibial osteoblast metabolism-related genes in aged laying hens. NCA: Normal calcium diet (3.57% Ca); LCA: Low calcium diet (2.08% Ca). SAL: Saline; LPS: Lipopolysaccharide. **(A)** *ALP*; **(B)** *Ocn*; **(C)** *FGF 23.* Data are represented as mean ± standard error of mean (n = 8). ^a,b^ Means with different alphabetical superscripts differ significantly, *p* < 0.05.

Figure 5 shows the mRNA expression of osteoblast metabolism-related genes in the tibia of laying hens. Feeding hens with the LCA diet upregulated (Figure 5A; *p* < 0.05) *ALP* expression compared to the NCA diet. However, hens injected with LPS showed a significant downregulation (Figure 5A; *p* < 0.05) in ALP expression levels compared to those injected with SAL.

The mRNA expression of *Ocn* was significantly influenced by the effect of diet × injection interaction (Figure 5B; *p* < 0.05). It was observed that the LCA + LPS group had a higher *Ocn* expression compared to the other treatment groups. However, the mRNA expression of *FGF23* was not influenced by the diet, injection or diet × injection interaction (Figure 5C; *p >* 0.05).

### 3.6 mRNA expression of tibial osteoclast metabolism-related genes

**FIGURE 6:**
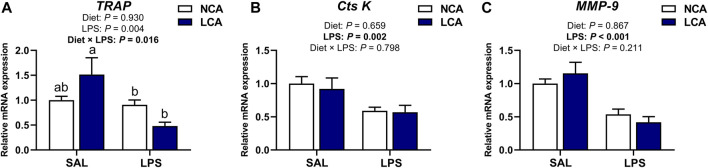
Effects of dietary calcium levels and LPS challenge on the relative mRNA expression of tibial osteoclast metabolism-related genes in aged laying hens. NCA: Normal calcium diet (3.57% Ca); LCA: Low calcium diet (2.08% Ca). SAL: Saline; LPS: Lipopolysaccharide. **(A)** *TRAP*; **(B)** *Cts K*; **(C)** *MMP-9*. Data are represented as mean ± standard error of mean (n = 8). ^a,b^ Means with different alphabetical superscripts differ significantly, *p* < 0.05.

Figure 6 shows the mRNA expression of osteoclast metabolism-related genes in the tibia of laying hens. *TRAP* expression was changed by the interaction of diet × LPS (Figure 6A; *p <* 0.05) and the LCA + SAL group had an upregulated *TRAP* expression compared to the NCA + LPS and LCA + LPS groups.

The mRNA expression of Cathepsin K (***Cts K***) and matrix metallopeptidase 9 (***MMP-9***) were significantly affected by the main effect of injection. LPS injection downregulated the tibial expression of *Cts K* (Figure 6B; *p <* 0.01) and *MMP-9* (Figure 6C; *p <* 0.001) compared to the SAL-injected hens.

## 4 Discussion

The present study evaluated the effects of dietary Ca levels and immune challenge on the performance, egg quality, immune response, and bone quality of aged laying hens. Findings from this study revealed that the LPS challenge reduced hen-day egg production, deteriorated the eggshell quality, and induced an inflammatory response that altered bone immunity and homeostasis by suppressing osteoblasts and osteoclast activities in aged laying hens. Also, there was a significant interaction between low calcium diet and LPS challenge that induced the pro-inflammatory response of *IL-17* expression, as well as *Ocn* expression in the tibia of aged laying hens.

Several studies have shown that Ca supply is important for egg production and eggshell quality at various stages of lay. A considerable amount of Ca is required for shell formation since the eggshell constitutes −40% Ca. However, Ca availability is largely dependent on dietary supply, intestinal absorption, and bone metabolism (Zhao et al., 2020; Hofmann et al., 2021). In a previous study, feeding low calcium diets (1.5% Ca) lowered the body weight, feed consumption, and egg production of laying hens (Zhao et al., 2020). In line with the present study, the dietary trial before LPS challenge revealed that the LCA diet depreciated the eggshell quality indices (including the eggshell weight, percentage, strength, and thickness), especially at 88 weeks of age. To corroborate this, feeding with LCA diet beyond 88 weeks of age decreased several performance and egg quality indices such as the hen-day egg production, egg mass, eggshell weight, eggshell percentage, eggshell strength, and eggshell thickness of aged hens. Thus, laying performance and eggshell quality of laying hens severely deteriorated with decreased dietary Ca supply during the extended laying phase. This may occur due to the crucial role of Ca in regulating reproductive processes and egg formation. This finding is supported by a previous report which stated that eggshell quality severely declined in hens fed a low calcium diet (Jiang et al., 2013). However, the dietary treatment in the present study did not affect the serum concentrations of ionized Ca and P, suggesting that the circulating metabolites were not strictly dependent on dietary Ca levels. Similarly, Zhao et al. (Zhao et al., 2020) reported that a low calcium diet decreased eggshell quality but without any detectable influence on serum Ca and P levels. Thus, the result suggests that circulating ionized Ca and P levels are tightly regulated by various factors in laying hens beyond dietary change. Interestingly, there was an interactive effect whereby NCA- and LCA-fed hens challenged with LPS, and LCA-fed hens injected with SAL, exhibited decreased Ca content in eggshells. This indicates that to some extent, Ca deprivation alongside exposure to immune challenge could pose detrimental effects on egg production in aged laying hens.

In the present study, the immune challenge with LPS administration also caused severe alterations to the production performance and eggshell quality of aged hens. LPS injection decreased the average daily feed intake, laying rate, and average daily egg mass. This inversely caused a disproportionate increase in the feed-to-egg ratio due to the fewer and smaller eggs. This finding corroborates the previous report that laying hens subjected to LPS-induced immune stress had poor egg quality traits and production performance including low feed intake, decreased egg production rate, and high feed-to-egg ratio (Nie et al., 2018).

In another study, maternal stimulation with LPS administration diminished the laying rate of hens and their offspring during the early and late laying stages (Liu et al., 2022). The depreciation in production performance during LPS challenge is credited to dysregulations in nutrient metabolism in order to eliminate potential threats from the body (Akbari MoghaddamKakhki and Kiarie, 2021). In the present study, LPS challenge also decreased the eggshell weight, eggshell percentage, eggshell thickness, and Ca and P content of the eggshell. Thus, exposure to immune stressors may alter the mineral metabolism and nutrient supply for eggshell formation. This can further aggravate eggshell deterioration in aged hens. Correspondingly, LPS administration was shown to decrease the eggshell percentage and eggshell weight of laying hens (Akbari MoghaddamKakhki and Kiarie, 2021). Importantly, egg production and eggshell quality are inextricably linked to Ca availability and bone metabolism in laying hens (Li et al., 2020; Zhao et al., 2020). Thus, bone homeostasis as influenced by Ca supply and immune challenge was further examined.

Bone homeostasis is achieved by a balance between bone resorption by osteoclasts and bone formation by osteoblasts (Tanaka et al., 2005). The bone mechanic parameter evaluated in the present study were unaffected while the Ca content of the tibia was even increased by LPS challenge, suggesting that the decreased bone Ca output. This observation was speculated to be related to the reduced egg production and in turn Ca deposit in eggshell. The H & E staining result indicated that LPS-challenged hens had more and larger adipocytes, suggesting that the connective tissue was dominated by adipocytes in LPS-hens. This suggested that bone health deteriorated by LPS challenge in aged laying hens. The conclusion should be explained with caution as the quantification analysis was not conducted in the histological observation. In previous studies, low-calcium diet or age reduces bone quality (Jiang et al., 2019; Huang et al., 2020). In this study, however, the detrimental effect of low-calcium diet on bone parameters was not observed. The relative short experimental period and poor laying performance should be responsible for the different observations.

Cytokines are important in regulating bone homeostasis and immune functions (Horowitz, 1998). The differentiation and activation of the bone cells including the osteoclasts and osteoblasts are driven by cytokines and immune cells (Li et al., 2021). Thus, the role of various cytokines on bone metabolism during LPS-induced immune challenge was investigated. Administration of LPS upregulated the mRNA expression of *TNF-α, IL-1β*, and *IL-6* in the spleen thus validating the successful stimulation of the pro-inflammatory response and immune system activation in laying hens (Mireles et al., 2005; Bai et al., 2019). Similarly, the mRNA expression of *TNF-α*, *IL-17, IL-1β*, *IL-6*, *IL-10,* and *IFN-γ* was increased by LPS challenge in the tibia of aged hens. This indicates the establishment of both pro- and anti-inflammatory milieu within the bone matrix. This finding is in line with Roodman (Roodman, 1993), who explained that it was crucial to maintain an adequate pool of cytokines and immune cells for bone homeostasis. This is because the relative proportions of these factors in the bone microenvironment play a critical role in regulating the activity of osteoblasts and osteoclasts. In addition, it was found that the mRNA expression of *TNF-α*, *IL-17,* and *IFN-γ* tended to increase in the tibia of LCA-fed hens challenged with LPS injection. In a recent review, these immune cytokines (TNF-α, IL-17, and IFN-γ) were identified as particularly significant in stimulating bone resorption and suppressing bone formation, which ultimately leads to inflammatory bone loss (Li et al., 2021). Thus, it is evident that low Ca supply during immune challenge would exacerbate the incidence of bone loss and dysregulate bone homeostasis in aged laying hens.

In the present study, dietary Ca levels did not exert remarkable changes to the bone parameters and immune cytokine production, however, the serum ALP level was increased by LCA diet, whereas, decreased with LPS challenge. This was further validated with the mRNA expression analysis of osteoblast metabolism-related genes. The findings revealed that LCA diet upregulated the expression of *ALP* and *Ocn* expression in the tibia of aged laying hens. Within bone tissue, ALP is synthesized by osteoblasts and functions in osteoid formation and bone mineralization (Kumar et al., 2018). ALP is highly expressed in mineralized tissue and the serum ALP can be used as an index to examine bone formation, bone turnover, and metabolism (Zhan et al., 2019; Vimalraj, 2020). In addition, Ocn is a non-collagenous protein abundant in the bone matrix (Dirckx et al., 2019). Produced by osteoblasts, Ocn plays an important role in bone formation and mineralization (Wei et al., 2021). Therefore, coinciding with the findings of the bone microstructure, the increased ALP activity and expression of both *ALP* and *Ocn* by LCA diet indicated an increased osteoblast activity, extensive bone mineralization, and abnormal bone metabolism in aged laying hens. Additionally, *Ocn* expression was elevated in LCA-fed hens exposed to LPS challenge, revealing the role of immune stress in aggravating disorders of bone metabolism. Conversely, the administration of LPS caused an inhibitory reaction by decreasing ALP activity and gene expression. This suggests a reduction in osteoblast activity, which can potentially destabilize the bone matrix and phosphate supply for bone minerals during immune challenge (Blair et al., 2017). In primary mouse calvarial osteoblast cells, LPS stimulation suppressed osteoblast differentiation via inhibiting ALP activity and the expression of *Ocn* (Cai et al., 2019). It was also observed that the LPS challenge elicited opposing effects on the *ALP* and *Ocn* expression. These changes cannot be fully explained but may reflect metabolic adaptations during immune stress to achieve bone homeostasis. Although the osteocytes and osteoblasts are a significant source of circulatory FGF23 (Yoshiko et al., 2007), treatment with low Ca and LPS challenge did not exert profound changes to the bone *FGF23* expression in aged hens. An examination of osteoclast metabolism-related genes revealed that immune stimulation with LPS inhibited the mRNA expression of *TRAP*, *CtsK*, and *MMP-9.* TRAP is an enzyme that can degrade skeletal phosphoproteins and it is widely used as a marker of osteoclast activity (Hayman, 2008). Cts K can degrade type I collagen in osteoclastic bone resorption (Wilson et al., 2009), and MMP-9 is known to mediate bone resorption by cleaving type I and IV collagen from the bone organic matrix (Guo et al., 2019). The tibial mRNA expression of *TRAP*, *Cts K*, and *MMP-9* was downregulated with LPS challenge. These findings suggest that LPS challenge impaired bone osteoclast activity and bone resorption capacity in aged laying hens. Noteworthy, a limitation of the present study was that few hens were used per replicate to assess their physiological responses during LPS challenge. However, this was necessary to meet the experimental purpose and adapt to practical conditions with the intent of evaluating the immune responses of birds to LPS challenge. It is suggested that a large number of hens should be adopted for further studies to validate findings and meet commercial production standards. Moreover, in the present study, the experimental diet was designed to reduce Ca level with fixed P level, resulting in a changed Ca to P ratio. Hence, the result cannot be ascribed to low-calcium diet solely. The interaction of altered Ca/P ratio and LPS-challenge needs to be investigated further.

In conclusion, both dietary Ca levels and immune challenge are important factors to consider in aged laying hens, especially during the extended laying period. The findings from this study demonstrate that LPS challenge and/or LCA diet would negatively affects traits related to the production performance and eggshell quality of aged laying hens, and also significantly alter bone homeostasis. The LCA diet altered osteoblast activity, bone metabolism, and reduced eggshell quality. Importantly, LPS-induced immune responses dysregulated osteoblastic mineralization and inhibited osteoclast bone resorption capacity. LPS challenge may have altered bone metabolism via increasing bone Ca deposits, with a significant reduction in Ca output from the bone to meet the Ca needs for eggshell formation in aged hens. Therefore, further studies on the underlying mechanisms of dietary Ca on the osteo-immunity of aged laying hens are recommended to harness the production potentials of hens during extended lay.

## Data Availability

The datasets presented in this study can be found in online repositories. The names of the repository/repositories and accession number(s) can be found below: https://www.ncbi.nlm.nih.gov/genbank/; NM_204524.1; NM_204628.1; NM_204460.1; NM_001004414.2; NM_205149.1; NM_205360.1; NM_205387.4; XM_425663.4; XM_015302697.2; NM_204971.2; NM_204667.1; NM_204305.1.

## Abbreviations

ALP, alkaline phosphatase; BMD, bone mineral density; Ca, calcium; Cts K, Cathepsin K; FGF23, fibroblast growth factor 23; GAPDH, glyceraldehyde-phosphate dehydrogenase; IL-1, Interleukin-1; IL-6, Interleukin-6; IL-10, Interleukin-10; IL-17, Interleukin-17; IFN-γ, interferon-gamma; LPS, lipopolysaccharide; MMP-9, matrix metallopeptidase 9; N, Newton; Ocn, osteocalcin; P, phosphorus; TNF-α, tumor necrosis factor-alpha; TRAP, tartrate-resistant acid phosphatase.

## References

[B1] Akbari MoghaddamKakhki R. Kiarie E. G. (2021). Effect of *Escherichia coli* lipopolysaccharide challenge on eggshell, tibia, and keel bone attributes in ISA brown hens exposed to dietary n-3 fatty acids prior to onset of lay. Poult. Sci. 100, 101431. 10.1016/j.psj.2021.101431 34607148 PMC8493573

[B2] Amarasekara D. S. Yu J. Rho J. (2015). Bone loss triggered by the cytokine network in inflammatory autoimmune diseases. J. Immunol. Res. 2015, 832127. 10.1155/2015/832127 26065006 PMC4434203

[B3] Bai W. Q. Zhang K. Y. Ding X. M. Bai S. P. Wang J. P. Peng H. W. (2019). High dietary energy content increases inflammatory markers after lipopolysaccharide challenge in meat ducks. Poult. Sci. 98, 164–171. 10.3382/ps/pey380 30137491

[B4] Bielke L. R. Hargis B. M. Latorre J. D. (2017). Impact of enteric health and mucosal permeability on skeletal health and lameness in poultry. Adv. Exp. Med. Biol. 1033, 185–197. 10.1007/978-3-319-66653-2_9 29101656

[B5] Blair H. C. Larrouture Q. C. Li Y. Lin H. Beer-Stoltz D. Liu L. (2017). Osteoblast differentiation and bone matrix formation *in vivo* and *in vitro* . Tissue Eng. Part B Rev. 23, 268–280. 10.1089/ten.TEB.2016.0454 27846781 PMC5467150

[B6] Cai P. Cai T. Li X. Fan L. Chen G. Yu B. (2019). Herbacetin treatment remitted LPS induced inhibition of osteoblast differentiation through blocking AKT/NF-κB signaling pathway. Am. J. Transl. Res. 11, 865–874. 30899386 PMC6413242

[B7] Close B. Banister K. Baumans V. Bernoth E. M. Bromage N. Bunyan J. (1997). Recommendations for euthanasia of experimental animals: Part 2. DGXT of the European Commission. Lab. Anim. 31, 1–32. 10.1258/002367797780600297 9121105

[B8] Dar H. Y. Azam Z. Anupam R. Mondal R. K. Srivastava R. K. (2018). Osteoimmunology: the nexus between bone and immune system. Front. Biosci. Landmark Ed. 23, 464–492. 10.2741/4600 28930556

[B9] Dirckx N. Moorer M. C. Clemens T. L. Riddle R. C. (2019). The role of osteoblasts in energy homeostasis. Nat. Rev. Endocrinol. 15, 651–665. 10.1038/s41574-019-0246-y 31462768 PMC6958555

[B10] Gu Y. F. Chen Y. P. Jin R. Wang C. Wen C. Zhou Y. M. (2021). A comparison of intestinal integrity, digestive function, and egg quality in laying hens with different ages. Poult. Sci. 100, 100949. 10.1016/j.psj.2020.12.046 33652523 PMC7936206

[B11] Guo J. Zeng X. Miao J. Liu C. Wei F. Liu D. (2019). MiRNA-218 regulates osteoclast differentiation and inflammation response in periodontitis rats through mmp9. Cell. Microbiol. 21, e12979. 10.1111/cmi.12979 30444938

[B12] Hayman A. R. (2008). Tartrate-resistant acid phosphatase (TRAP) and the osteoclast/immune cell dichotomy. Autoimmunity 41, 218–223. 10.1080/08916930701694667 18365835

[B13] Hofmann T. Schmucker S. Sommerfeld V. Huber K. Rodehutscord M. Stefanski V. (2021). Immunomodulatory effects of dietary phosphorus and calcium in two strains of laying hens. Anim. (Basel) 11, 129. 10.3390/ani11010129 PMC7826506 33430096

[B14] Horowitz M. C. (1998). The role of cytokines in bone remodeling. J. Clin. Densitom. 1, 187–198. 10.1385/jcd:1:2:187

[B15] Huang J. Tong X. F. Yu Z. W. Hu Y. P. Zhang L. Liu Y. (2020). Dietary supplementation of total flavonoids from Rhizoma Drynariae improves bone health in older caged laying hens. Poult. Sci. 99, 5047–5054. 10.1016/j.psj.2020.06.057 32988541 PMC7598317

[B16] Jiang S. Cui L. Shi C. Ke X. Luo J. Hou J. (2013). Effects of dietary energy and calcium levels on performance, egg shell quality and bone metabolism in hens. Vet. J. 198, 252–258. 10.1016/j.tvjl.2013.07.017 24054908

[B17] Jiang S. Wu X. L. Jin M. L. Wang X. Z. Tang Q. Sun Y. X. (2019). Pathophysiological characteristics and gene transcriptional profiling of bone microstructure in a low calcium diet fed laying hens. Poult. Sci. 98, 4359–4368. 10.3382/ps/pez271 31073614

[B18] Kim Y. G. Park J. W. Lee J. M. Suh J. Y. Lee J. K. Chang B. S. (2014). IL-17 inhibits osteoblast differentiation and bone regeneration in rat. Arch. Oral. Biol. 59, 897–905. 10.1016/j.archoralbio.2014.05.009 24907519

[B19] Knothe Tate M. L. Adamson J. R. Tami A. E. Bauer T. W. (2004). The osteocyte. Int. J. Biochem. Cell. Biol. 36, 1–8. 10.1016/s1357-2725(03)00241-3 14592527

[B20] Kumar S. Maurya R. (2018). “Chapter 8 - plant drugs in the treatment of osteoporosis,” in Natural products and drug discovery. Editors Mandal S. C. Mandal V. Konishi T. (Elsevier), 179–212.

[B21] Ledur M. C. Fairfull R. W. McMillan I. Asseltine L. (2000). Genetic effects of aging on egg production traits in the first laying cycle of White Leghorn strains and strain crosses. Poult. Sci. 79, 296–304. 10.1093/ps/79.3.296 10735193

[B22] Li N. Fu L. Li Z. Ke Y. Wang Y. Wu J. (2021). The role of immune microenvironment in maxillofacial bone Homeostasis. Front. Dent. Med. 2, 780973. 10.3389/fdmed.2021.780973

[B23] Li T. Xing G. Shao Y. Zhang L. Li S. Lu L. (2020). Dietary calcium or phosphorus deficiency impairs the bone development by regulating related calcium or phosphorus metabolic utilization parameters of broilers. Poult. Sci. 99, 3207–3214. 10.1016/j.psj.2020.01.028 32475457 PMC7597650

[B24] Liu L. Wang D. Li X. Adetula A. A. Khan A. Zhang B. (2022). Long-lasting effects of lipopolysaccharide on the reproduction and splenic transcriptome of hens and their offspring. Ecotoxicol. Environ. Saf. 237, 113527. 10.1016/j.ecoenv.2022.113527 35453024

[B25] Mercier F. E. Ragu C. Scadden D. T. (2011). The bone marrow at the crossroads of blood and immunity. Nat. Rev. Immunol. 12, 49–60. 10.1038/nri3132 22193770 PMC4013788

[B26] Mireles A. J. Kim S. M. Klasing K. C. (2005). An acute inflammatory response alters bone homeostasis, body composition, and the humoral immune response of broiler chickens. Poult. Sci. 84, 553–560. 10.1093/ps/84.4.553 15844811

[B27] Molnar A. Maertens L. Ampe B. Buyse J. Zoons J. Delezie E. (2017). Supplementation of fine and coarse limestone in different ratios in a split feeding system: effects on performance, egg quality, and bone strength in old laying hens. Poult. Sci. 96, 1659–1671. 10.3382/ps/pew424 27920197

[B28] Molnar A. Maertens L. Ampe B. Buyse J. Zoons J. Delezie E. (2018). Effect of different split-feeding treatments on performance, egg quality, and bone quality of individually housed aged laying hens. Poult. Sci. 97, 88–101. 10.3382/ps/pex255 29077907

[B29] Nie W. Wang B. Gao J. Guo Y. Wang Z. (2018). Effects of dietary phosphorous supplementation on laying performance, egg quality, bone health and immune responses of laying hens challenged with *Escherichia coli* lipopolysaccharide. J. Anim. Sci. Biotechnol. 9, 53. 10.1186/s40104-018-0271-z 30123501 PMC6088422

[B30] Roodman G. D. (1993). Role of cytokines in the regulation of bone resorption. Calcif. tissue Int. 53, S94–S98. 10.1007/BF01673412 8275387

[B31] Song M. Jiao H. Zhao J. Zhao X. Li H. Wang P. (2022). Dietary supplementation of calcium propionate and calcium butyrate improves eggshell quality of laying hens in the late phase of production. J. Poult. Sci. 59, 64–74. 10.2141/jpsa.0200127 35125914 PMC8791774

[B32] Sun M. Zhao J. Wang X. Jiao H. Lin H. (2020). Use of encapsulated L-lysine-HCl and DL-methionine improves postprandial amino acid balance in laying hens. J. Anim. Sci. 98, skaa315. 10.1093/jas/skaa315 32954399 PMC7759752

[B33] Takayanagi H. (2007). Osteoimmunology: shared mechanisms and crosstalk between the immune and bone systems. Nat. Rev. Immunol. 7, 292–304. 10.1038/nri2062 17380158

[B34] Tanaka Y. Nakayamada S. Okada Y. (2005). Osteoblasts and osteoclasts in bone remodeling and inflammation. Curr. Drug Targets Inflamm. Allergy 4, 325–328. 10.2174/1568010054022015 16101541

[B35] Tsukasaki M. Takayanagi H. (2019). Osteoimmunology: evolving concepts in bone-immune interactions in health and disease. Nat. Rev. Immunol. 19, 626–642. 10.1038/s41577-019-0178-8 31186549

[B36] Vimalraj S. (2020). Alkaline phosphatase: structure, expression and its function in bone mineralization. Gene 754, 144855. 10.1016/j.gene.2020.144855 32522695

[B37] Walsh M. C. Takegahara N. Kim H. Choi Y. (2018). Updating osteoimmunology: regulation of bone cells by innate and adaptive immunity. Nat. Rev. Rheumatol. 14, 146–156. 10.1038/nrrheum.2017.213 29323344 PMC5821527

[B38] Wei H. Chen Y. Nian H. Wang J. Liu Y. Wang J. (2021). Abnormal bone metabolism may be a primary causative factor of keel bone fractures in laying hens. Anim. (Basel) 11, 3133. 10.3390/ani11113133 PMC8614394 34827866

[B39] Whitehead C. C. (2004). Overview of bone biology in the egg-laying hen. Poult. Sci. 83, 193–199. 10.1093/ps/83.2.193 14979569

[B40] Wilson S. R. Peters C. Saftig P. Brömme D. (2009). Cathepsin K activity-dependent regulation of osteoclast actin ring formation and bone resorption. J. Biol. Chem. 284, 2584–2592. 10.1074/jbc.M805280200 19028686 PMC2629117

[B41] Xin Q. Ma N. Jiao H. Wang X. Li H. Zhou Y. (2022). Dietary energy and protein levels during the prelay period on production performance, egg quality, expression of genes in hypothalamus-pituitary-ovary axis, and bone parameters in aged laying hens. Front. Physiol. 13, 887381. 10.3389/fphys.2022.887381 35574467 PMC9096247

[B42] Yoshiko Y. Wang H. Minamizaki T. Ijuin C. Yamamoto R. Suemune S. (2007). Mineralized tissue cells are a principal source of FGF23. Bone 40, 1565–1573. 10.1016/j.bone.2007.01.017 17350357

[B43] Zhan X. Yang Y. Chen Y. Wei X. Xiao J. Zhang L. (2019). Serum alkaline phosphatase levels correlate with long-term mortality solely in peritoneal dialysis patients with residual renal function. Ren. Fail 41, 718–725. 10.1080/0886022X.2019.1646662 31409217 PMC6713195

[B44] Zhang F. Tanaka H. Kawato T. Kitami S. Nakai K. Motohashi M. (2011). Interleukin-17A induces cathepsin K and MMP-9 expression in osteoclasts via celecoxib-blocked prostaglandin E2 in osteoblasts. Biochimie 93, 296–305. 10.1016/j.biochi.2010.10.001 20937352

[B45] Zhao S. C. Teng X. Q. Xu D. L. Chi X. Ge M. Xu S. W. (2020). Influences of low level of dietary calcium on bone characters in laying hens. Poult. Sci. 99, 7084–7091. 10.1016/j.psj.2020.08.057 33248625 PMC7704722

